# Pain Severity During Hysteroscopy by GUBBINI System in Local Anesthesia: Covariance Analysis of Treatment and Effects, Including Patient Emotional State

**DOI:** 10.3390/jcm13206217

**Published:** 2024-10-18

**Authors:** Karolina Chmaj-Wierzchowska, Aleksandra Jasielska, Katarzyna Wszołek, Katarzyna Tomczyk, Agnieszka Lach, Adrian Mruczyński, Martyna Niegłos, Aleksandra Wilczyńska, Kinga Bednarek, Maciej Wilczak

**Affiliations:** 1 Department of Maternal and Child Health, Poznan University of Medical Sciences, 61-701 Poznan, Polandkatarzyna.zielin@gmail.com (K.T.); lach.agnieszka95@gmail.com (A.L.); adrianmrucz@gmail.com (A.M.); marticzku@gmail.com (M.N.); aleksandra.juckiewicz@gamil.com (A.W.); kinga.bednarek88@gmail.com (K.B.); mwil@ump.edu.pl (M.W.); 2 Faculty of Psychology and Cognitive Science, Adam Mickiewicz University, 61-712 Poznan, Poland

**Keywords:** minihysteroscopy, pain, alexithymia

## Abstract

Pain accompanying medical procedures can be considered in the “mind-body” problem of accounting for and describing the relationship between mental and physical processes (psyche and soma). **Background/Objectives**: The purpose of this study is to evaluate the severity of pain among patients undergoing a minihysteroscopy procedure under local anesthesia using the “GUBBINI SYSTEM” (GUBBINI Mini Hystero-Resectoscope; Tontarra Medizintechnik, Tuttlingen, Germany) and to assess the association of various covariates with pain during the procedure, including patient emotional state. **Methods:** This study included 171 patients admitted to the Center for Hysteroscopy under Local Anesthesia at the Heliodor Święcicki Gynecological and Obstetrical Clinical Hospital of the Karol Marcinkowski Medical University in Poznań, Poland, for hysteroscopic treatment under local anesthesia (paracervical, using lignocaine). The Center for Hysteroscopy is the first certified “CENTER OF EXCELLENCE” of The International Society for Gynecologic Endoscopy (ISGE) in Poland. **Results**: A positive relationship was observed between alexithymia and its trait of difficulty identifying emotions and pain, as well as between perceived pain and one of the deficits of emotional processing—signs of unprocessed emotion. **Conclusions**: In conclusion, before the hysteroscopy, adequate information and counseling related to the procedure can effectively reduce the pain and anxiety levels of the women, and nurses can navigate this stressful process. Providing education and counseling to all women undergoing hysteroscopy, and explaining the procedure in detail, should be the preferred approach.

## 1. Introduction

Hysteroscopic procedures are minimally invasive and allow for the assessment of the uterine cavity (diagnostic hysteroscopy) and treatment of intrauterine lesions (operative hysteroscopy). Hysteroscopy is widely regarded as the gold standard for evaluating pathology in both premenopausal and postmenopausal patients, as well as for assessing suspected uterine cavity pathology in infertile patients [1], replacing uterine cavity curettage under general anesthesia. Currently, there is significant emphasis on minihysteroscopy, also known as office or outpatient hysteroscopy. The “see and treat” approach has reduced the distinction between diagnostic and operative procedures [2]. Minihysteroscopy allows for the procedure to be performed without dilating the cervical canal, reducing treatment costs and shortening hospitalization and recovery times [3,4].

Minihysteroscopy is performed under local anesthesia, with the patient remaining conscious during the procedure [5,6]. Therefore, providing emotional support (“voice anesthesia”) is recommended to reassure and engage the patient in the therapeutic process [2]. However, minihysteroscopy often induces significant anxiety, increasing the likelihood of intolerance to the procedure. High levels of anxiety, particularly related to pain rather than the intervention itself, predict a patient’s preference for general anesthesia in future procedures [7]. The use of smaller-diameter hysteroscopes does not guarantee a pain-free procedure in all cases [8,9,10]. According to international research, 1.3%–5.2% of hysteroscopy procedures may be impossible to complete due to reported pain [2]. Pain severity may be influenced by technical factors, operator experience, procedure duration, reproductive organ abnormalities, and the patient’s psychological profile [11]. Anxiety remains a common issue in hysteroscopy, prompting the use of both pharmacological and nonpharmacological methods to alleviate intraoperative and postoperative pain in minihysteroscopy [12].

Pain is defined as “an unpleasant sensory and emotional experience associated with, or resembling that associated with, actual or potential tissue damage” (The International Association for the Study of Pain) and is widely recognized as a multidimensional experience influenced by biopsychosocial factors [13]. Pain accompanying medical procedures can be considered within the framework of the “mind-body” problem, which explores the relationship between mental and physical processes (psyche and soma) [14]. In the context of psychopathology, two central questions emerge from this mind–body issue: which sphere takes precedence in the genesis and development of illness, and how does each sphere affect the other [14].

Research on the mental functioning of people experiencing pain shows that greater pain intensity is associated with specific affective factors, such as emotional stress, limited emotional awareness, and difficulties in emotional expression and processing [15]. For instance, emotions influence both pain perception and the cortical processing of pain [16,17,18]. Studies on the emotion–pain relationship have demonstrated that negative emotions can heighten pain perception in healthy individuals. The ability to regulate emotions during painful experiences is linked to the effective reduction of both the affective and sensory dimensions of pain [19]. Emotion regulation is considered a clinically significant factor in effective pain management [20,21,22,23].

The purpose of this study is to evaluate the severity of pain among patients undergoing a minihysteroscopy procedure under local anesthesia using the “GUBBINI SYSTEM” (GUBBINI Mini Hystero-Resectoscope; Tontarra Medizintechnik, Tuttlingen, Germany) and to assess the association of various covariates with pain during the procedure, including patient emotional state.

## 2. Materials and Methods

This study included patients admitted to the Center for Hysteroscopy under Local Anesthesia at the Heliodor Święcicki Gynecological and Obstetrical Clinical Hospital, part of the Karol Marcinkowski Medical University in Poznań, Poland, for hysteroscopic treatment under local anesthesia (paracervical, using lignocaine). The Center for Hysteroscopy is the first certified “CENTER OF EXCELLENCE” of the International Society for Gynecologic Endoscopy (ISGE) in Poland.

The criteria for qualifying for the procedure included the presence of abnormal uterine bleeding, changes in the uterine cavity such as endometrial polyps or submucosal myomas, endometrial hyperplasia, and endometrial hypertrophy. Patients with no history of sensitivity to lignocaine and ketoprofen, who were in phase I of the menstrual cycle or postmenopausal, were included. Exclusion criteria were heavy genital tract bleeding, inflammation of the vagina and/or cervix, or pregnancy.

A medical history was collected, including details on age, weight, height, past surgeries, presence of drug allergies, number and types of previous deliveries, number of miscarriages, and history of cervical and endometrial procedures, along with general health. After undergoing a gynecological examination and transvaginal ultrasound, patients provided informed consent for the hysteroscopic procedure.

An original survey questionnaire served as the research tool. In addition to questions about education, place of residence, marital status, and professional situation, patients assessed whether they experienced pain during sexual intercourse, bowel movements, and/or micturition, as well as pain in daily life and symptoms of depression. The following scales were also used: the Beck Depression Scale, the Toronto Alexithymia Scale (TAS-20), and the Emotional Processing Scale (EPS). The Beck Depression Scale was used for self-assessment of well-being. Its scores provide an indication, though not a diagnosis, of depression: 0–11 points indicate no depression (temporary mood decline due to current life events), 12–19 points indicate mild depression, and 20–25 points indicate moderate depression. The Toronto Alexithymia Scale (TAS-20) [24,25] measures the intensity of alexithymia, defined as the inability to express, describe, or distinguish among one’s emotions. It consists of 20 items that assess three factors aligned with the theoretical basis of alexithymia: (1) Difficulty Identifying Feelings, (2) Difficulty Describing Feelings, and (3) External Thinking Style. The questionnaire is designed for the general population, with respondents rating each item on a 5-point scale from 1 (completely disagree) to 5 (completely agree). The total alexithymia score is the sum of responses to all 20 items. Subscale scores are calculated similarly. TAS-20 cutoff scores are as follows: a total score of 51 or less indicates no alexithymia, 61 or more indicates current alexithymia, and scores from 52 to 60 suggest possible alexithymia. The Emotional Processing Scale (EPS) [26] measures deficits in emotional processing. It comprises 25 items divided into five subscales that address distortions in emotional processing: suppression, lack of processing, lack of regulation, avoidance, and poor experience of emotions. Higher scores on each subscale indicate greater deficits in that area, while the total score reflects the overall difficulty in regulating emotional arousal. Respondents are asked to recall an event from the past week and identify the most strongly felt negative and positive emotions. They then rate their feelings and behaviors in response to 25 statements on a scale from 0 (completely disagree) to 9 (fully agree).

The questionnaires were completed by patients 60 min before the procedure. The study participants were divided into two groups based on the type of procedure (diagnostic hysteroscopy vs. operative hysteroscopy). During the procedures, patients remained conscious and were able to observe the procedure in real time on a monitor.

Description of the procedure. Thirty minutes before the start of the hysteroscopy procedure, each qualified patient received 100 mg of ketoprofen intravenously. Throughout the procedure, vital signs, including heart rate, blood pressure, oxygen saturation, and respiratory rate, were continuously monitored. Ten minutes before the insertion of the minihysteroscope into the cervical canal, 10 mL of a 0.1% lignocaine solution was administered pericervically at two points (10 mL at the 4 o’clock position and 10 mL at the 8 o’clock position, where no large blood vessels are located, allowing for safe local anesthesia). A needle with an insertion depth of approximately 2 mm was used (Hystero-Block). The hysteroscopy procedure was performed using vaginoscopy, without the insertion of a speculum or culotte. The procedure was conducted by three operators with comparable experience (seven specialists, each performing approximately 800 procedures per year). Patients were positioned on a treatment table in the typical gynecological examination position. A 0.9% NaCl solution was used as the distension medium, with continuous flow and a pressure of 120 mmHg to dilate the uterine cavity. The procedure was carried out using the GUBBINI Mini Hystero-Resectoscope system. After the procedure, patients were asked to verbally assess their pain during hysteroscopy using a Visual Analog Scale (VAS). Pain levels were categorized as follows: 0–3 points for mild pain, 4–7 points for moderate pain, and 8–10 points for severe pain. If a patient rated their pain above 4 points and requested additional pain relief, 200 mg of ibuprofen was administered intravenously.

Data analysis was performed using jamovi (version 2.3). Both descriptive and analytical statistics were applied. For the analytical component, ANCOVA, the Kruskal–Wallis test, and the Mann–Whitney *U* test were used. The level of statistical significance was set at *p* < 0.05.

## 3. Results

The study cohort comprised 171 patients admitted to the Center for Hysteroscopy under Local Anesthesia, for hysteroscopic treatment, aged 21 years or older.

### 3.1. Characteristics of the Study Group

Characteristics of the whole study group divided into age, marital status, education, occupational status, place of residence, previous gynecological surgeries, and obstetric interview (natural childbirth and cesarean section) are presented in Table 1.

The characteristics of the entire study group, including depression reported by the patient during the medical interview, as well as depression, antidepressant use, and psychological or psychiatric treatment declared in the research questionnaire, about the type of hysteroscopy performed are presented in Table 2.

Other, i.e., comorbidities, includes hypertension, chronic ischemic heart disease, asthma, hypothyroidism, and hyperthyroidism.

The characteristics of the entire study group, including pain during intercourse, defecation, urination, and other daily activities as reported in the research questionnaire, about the type of hysteroscopy performed are presented in Table 3.

The characteristics of the whole study group including clinical diagnosis, type of hysteroscopy performed, and the need to use additional analgesics are presented in Table 4.

### 3.2. Diagnostic vs. Operative Hysteroscopy

The characteristics of the variables (age, weight, height, obstetric interview) divided into diagnostic vs. operative hysteroscopy are presented in Table 5.

1. Diagnostic hysteroscopy (*n* = 49).
2. Operative hysteroscopy (*n* = 122).

The Shapiro–Wilk test statistic indicates a normal distribution for the variable “height” in both groups and for the variable “lesion size in mm (subjective size during hysteroscopy)” in the group undergoing operative hysteroscopy, despite the small sample size. The distribution of the other variables is also consistent with a normal distribution.

### 3.3. VAS Perioperative Pain

An analysis using Spearman’s rho test examined the associations between medical procedure parameters and reported pain in the entire study group, as well as in two subgroups: diagnostic hysteroscopy and operative hysteroscopy (Table 6). A significant weak positive association was observed between lesion size in millimeters and reported pain in both the entire group and the subgroup of women undergoing operative hysteroscopy. In contrast, in the subgroup of women undergoing diagnostic hysteroscopy, the association was strongly negative. Additionally, a weak positive association was observed between reported pain and lesion size as observed on ultrasound in the subgroup of women undergoing operative hysteroscopy.

The Kruskal–Wallis test showed no differences in declared pain between groups with different diagnoses (Table 7). The Steel–Dwass–Critchlow–Fligner pair-wise comparisons were not significant either.

The Mann–Whitney *U* test showed no differences in declared pain between groups undergoing different types of procedures: diagnostic hysteroscopy vs. operative hysteroscopy (Table 8). The comparison of pain between premenopausal and postmenopausal women was nonsignificant (KW = 2.32; df = 1; *p* = 0.127).

### 3.4. Psychological Tests, Discussion, and Presentation: What Is Allowed

#### 3.4.1. Does Diagnosed Depression Affect a Higher Pain/VAS Score After Surgery and More Often Require Additional Medication After Surgery?

To test whether patients’ declaration of suffering from depression and the type of hysteroscopy influence their reported pain levels, a two-factor ANCOVA analysis of variance was conducted using a 2 (declaration of depression: yes, no) × 2 (type of hysteroscopy: diagnostic, operative), as shown in Table 9.

The results showed a statistically significant interaction effect *F* (1, 167) = 4.495; *p* < 0.05, *η*^2^ = 0.03. The results of the analysis are shown in Table 10.

Patients who reported suffering from depression experienced a stronger sensation of pain during operative hysteroscopy compared to those who underwent diagnostic hysteroscopy.

#### 3.4.2. Does Latent Depression Affect Higher Pain/VAS Scores after Surgery and More Often Require Additional Medication after Surgery?

Based on the Beck Depression Inventory (BDI) scores, four groups of patients were identified: those with no depressive symptoms, mild depression, moderate depression, and severe depression. The Kruskal–Wallis test showed no significant differences in reported pain among the groups with varying levels of depression (Table 11). Subsequently, the study participants were grouped into two categories: patients without depression and patients with depression. Again, no statistically significant difference in reported pain was found between these two groups.

To test whether BDI-indicated depression and the type of hysteroscopy influence patients’ reported pain, an ANCOVA analysis of variance was conducted using a 2 (BDI score: no depression, depression) × 2 (type of hysteroscopy: diagnostic, operative) design, which proved to be nonsignificant.

#### 3.4.3. How Does the Emotional Realm Covary with Pain/VAS After Surgery and More Often Require Additional Medication After Surgery?

For all variables except alexithymia, the Shapiro–Wilk test proved to be statistically significant, indicating that the distribution of these variables is skewed from a normal distribution (Table 12).

A positive relationship was observed between alexithymia and its trait of difficulty identifying emotions and pain, as well as between perceived pain and one of the deficits of emotional processing—signs of unprocessed emotion (Table 13).

## 4. Discussion

In medical procedures where the patient remains conscious, a key factor influencing the level of pain experienced—and thus the feasibility of completing the procedure—is the patient’s perceived fear of it [27,28]. Patient anxiety in medical settings is a well-documented phenomenon, often exemplified by the “white coat effect” [29]. In this context, it is important to emphasize interventions aimed at reducing anxiety, particularly among female patients [30]. Basic measures should include explaining the purpose and course of the procedure in clear, understandable language. Other significant factors include minimizing waiting times after admission, providing dignified and comfortable waiting conditions, maintaining communication with patients, offering real-time updates, and promptly addressing their needs [27,28,31,32]. In this regard, the presence and attitude of the midwife—both in preprocedural and postprocedural care—and the support of a second midwife during the procedure are vital. Mahmud et al. [33] analyzed the experiences of women undergoing outpatient hysteroscopy and found that respondents greatly appreciated the staff’s care and professionalism, which positively affected their overall perception of the procedure. Since women remain conscious during outpatient hysteroscopy, communication should not be limited to the preoperative phase but should continue throughout the procedure. Morgan et al. [34] studied women’s experiences and attitudes during hysteroscopy [34], finding that patients valued continuous updates about the procedure’s progress and what they could expect next. However, 7% of respondents reported negative experiences, mainly due to a lack of information about potential pain, rushed interactions with staff, and waiting in areas that heightened their anxiety [33].

In some countries, hysteroscopy is performed by nurses or midwives [35]. The experience of the healthcare professional performing the procedure, along with key aspects like cervical preparation, equipment size, and the volume and rate of fluid introduced into the uterine cavity, is undeniably important [27,28,31,32]. It is worth asking whether factors beyond these might influence the intensity of pain during the procedure. Lee et al. [36] found that patients with depression undergoing surgical interventions experienced postoperative pain more intensely and for longer durations. Regarding anxiety, some researchers suggest that the anxiety women feel before hysteroscopy is comparable to that of women awaiting major surgery under general anesthesia [37].

Pain is subjective, comprising emotional and cognitive components, which complicates its evaluation as a symptom. Pain tolerance and response vary among individuals. Although data on the covariability between emotional functioning and reported pain are limited, they are consistent. The observed correlation between difficulty identifying affective experiences—a core feature of alexithymia—and pain aligns with previous studies [38]. This corresponds with the position of Lumley et al. [15], who claim that “Clinical observations suggest that people with limited emotional awareness and verbalization may describe emotions in somatic terms, such as ‘my muscles are tight’ or ‘my stomach hurts’”. Lane et al. [15] proposed a neuroscience model of alexithymia, suggesting that difficulty differentiating between emotions and physical sensations, as well as processing emotions consciously (i.e., alexithymia), may lead to pain reports infused with emotional content. This idea is supported by the observed link between pain and unprocessed emotion. A patient undergoing surgery who experiences emotional and physiological hyperarousal may block emotional processing, resulting in an enhanced perception of pain as a manifestation of unprocessed emotion. Thus, pain may serve as an indicator of unprocessed emotion [26]. To understand the relationship between pain and emotional status, it is important to recognize the multidimensional nature of pain, where sensory mechanisms play only a part in processing this complex phenomenon. Despite the well-documented advantages of outpatient hysteroscopy, there remains some reluctance to adopt this approach. This procedure is thought to provoke anxiety in patients, affecting overall satisfaction. However, our study does not support these concerns. Proper patient preparation and perioperative care are essential for the procedure’s success. Addressing anxiety-inducing factors can improve patient compliance and enhance the diagnostic and therapeutic potential of the procedure. Clear pre-exam explanations can predispose patients to a less traumatic experience.

Nonpharmacological interventions to reduce anxiety during hysteroscopy are promising. These include thorough patient education, clear explanations of the procedure, outlining potential deviations, providing support during the procedure, employing a “no-touch” approach using vaginoscopy and minihysteroscopes, reducing preprocedural waiting time, and allowing patients to listen to music or engage in conversation [29,39,40]. A recent randomized trial in office hysteroscopy demonstrated that listening to music during the procedure may reduce pain, likely due to decreased anxiety [29]. This knowledge serves as a crucial starting point for pain management in clinical practice, particularly for procedures that do not require anesthesia.

## 5. Conclusions

In conclusion, we observed a positive relationship between alexithymia—specifically the trait of difficulty identifying emotions—and pain, as well as between perceived pain and unprocessed emotion. Therefore, providing adequate information and counseling before hysteroscopy can effectively reduce both pain and anxiety levels in patients. Nurses play a crucial role in navigating this stressful process. Comprehensive education and counseling for women undergoing hysteroscopy, with a detailed explanation of the procedure, should be standard practice.

## Figures and Tables

**Table 1:** Characteristics of the whole study group.

Variable	Category	Hysteroscopy	
		Diagnostic (*n* = 49) (%)	Operative (*n* = 122) (%)
Age	21–30 years	5 (10.20%)	16 (13.11%)
	31–40 years	19 (38.77%)	51 (41.80%)
	41–50 years	17 (34.69%)	37 (30.33%)
	51–60 years	4 (8.16%)	12 (9.84%)
	>61 years	4 (8.16%)	6 (4.92%)
Marital status	Maiden	10 (20.41%)	25 (20.49%)
	Married	29 (59.18%)	85 (69.67%)
	Widowed	1 (2.04%)	3 (2.46%)
	Single	9 (18.38%)	9 (7.38%)
Education	Primary	1 (2.04%)	1 (0.82%)
	Vocational	3 (6.12%)	7 (5.74%)
	Secondary	17 (34.69%)	33 (27.05%)
	Bachelor’s degree	4 (8.16%)	13 (10.66%)
	Higher	24 (48.98%)	68 (55.74%)
Occupational status	Retirement	4 (8.16%)	5 (4.10%)
	I am learning/studying	4 (8.16%)	5 (4.10%)
	I work physically	10 (20.41%)	28 (22.95%)
	I work mentally	31 (63.26%)	84 (68.85%)
Place of residence	Village < 2000 citizens	20 (40.82%)	45 (36.88%)
	City 2000–50,000 citizens	9 (18.38%)	21 (17.21%)
	City 50,000–200,000 citizens	5 (10.20%)	10 (8.20%)
	City 200,000–500,000 citizens	4 (8.16%)	5 (4.10%)
	City > 500,000 citizens	11 (22.45%)	41 (33.60%)
Previous gynecological surgeries	Cesarean section	6 (12.24%)	12 (9.84%)
	Surgeries other than gynecological	5 (10.20%)	35 (28.69%)
	Minor gynecological procedure (abrasio/hysteroscopy)	10 (20.41%)	20 (16.39%)
	Moderate gynecological procedure (laparoscopy/hysterolaparoscopy)	1 (2.04%)	2 (1.64%)
	Major gynecological surgery (laparotomy/laparoscopy)	3 (6.12%)	6 (4.92%)
	No	24 (48.98%)	47 (38.52%)
Natural childbirth	0	22 (44.89%)	42 (34.42%)
	1	4 (8.16%)	32 (26.22%)
	2	18 (36.73%)	40 (32.78%)
	3 and more	5 (10.20%)	8 (6.56%)
Cesarean section	0	42 (85.71%)	107 (87.70%)
	1	5 (10.20%)	11 (9.02%)
	2	2 (4.08%)	4 (3.28%)

**Table 2:** Characteristics of the whole study group including depression.

		Hysteroscopy	
		Diagnostic (*n* = 49) (%)	Operative (*n* = 122) (%)
Diseases	Other	26 (53.06%)	59 (48.36%)
	Other, depression	3 (6.12%)	7 (5.74%)
	Other, depression, neurosis	1 (2.04%)	1 (0.82%)
	Other, migraine	2 (4.08%)	3 (2.46%)
	Other, anxiety disorders	1 (2.04%)	4 (3.28%)
	Migraine	1 (2.04%)	2 (1.64%)
	Migraine, depression	0 (0%)	3 (2.46%)
	Depression	0 (0%)	2 (1.64%)
	No	15 (30.61%)	41 (33.60%)
Depression	No	44 (89.79%)	104 (85.25%)
	Yes	5 (10.20%)	18 (14.75%)
Antidepressants	No	48 (97.96%)	106 (86.88%)
	Yes	1 (2.04%)	16 (13.11%)
Psychiatric control	Yes	2 (4.08%)	17 (13.93%)
	No	47 (95.91%)	105 (86.06%)
Psychological control	Yes	6 (4.08%)	8 (6.56%)
	No	43 (87.75%)	114 (93.44%)

**Table 3:** Characteristics of the whole study group including pain.

		Hysteroscopy	
		Diagnostic (*n* = 49) (%)	Operative (*n* = 122) (%)
Pain during intercourse	Yes	12 (24.49%)	24 (19.67%)
	No	37 (75.51%)	98 (80.33%)
Pain during defecation	Yes	1 (2.04%)	1 (0.82%)
	No	48 (97.96%)	121 (99.18%)
Pain during urination	Yes	0 (0%)	2 (1.64%)
	No	49 (100%)	120 (98.36%)
Pain during other activities	Yes	1 (2.04%)	5 (4.10%)
	No	48 (97.96%)	117 (95.90%)

**Table 4:** Characteristics of the whole study group including clinical diagnosis, type of hysteroscopy performed, and additional analgesics after hysteroscopy.

		Hysteroscopy	
		Diagnostic (*n* = 49) (%)	Operative (*n* = 122) (%)
Diagnosis	Polyp	2 (4.08%)	91 (74.59%)
	Hypertrophy/abnormal bleeding	6 (12.24%)	13 (10.66%)
	Myomas	29 (59.18%)	14 (11.47%)
	Infertility/miscarriages	12 (24.49%)	4 (3.28%)
Number of diagnoses	1	27 (55.10%)	92 (75.41%)
	>1	22 (44.90%)	30 (24.60%)
Procedure	Operative hysteroscopy; endometrial polyp resection	0 (0%)	97 (79.50%)
	Operative hysteroscopy; uterine myoma resection	0 (0%)	15 (12.29%)
	Operative hysteroscopy; polypoid endometrial hyperplasia resection	0 (0%)	10 (8.20%)
	Diagnostic hysteroscopy; endometrial biopsy	41 (83.67%)	0 (0%)
	Diagnostic hysteroscopy; biopsy of the endometrium, biopsy of the cervical canal, cervical disc specimens	8 (16.33%)	0 (0%)
Additional analgesic	Yes	4 (8.16%)	21 (17.21%)
	No	45 (91.84%)	101 (82.77%)

**Table 5:** Characteristics of the study group.

Variable	Procedure	*M*	SD	Min.	Max.	*W*	*p*
Age	1	42.816	10.996	22	77	0.946	0.026
	2	40.951	9.747	21	68	0.964	0.002
Weight	1	69.531	13.692	49	108	0.944	0.021
	2	71.959	16.913	48	138	0.905	<0.001
Height	1	166.306	6.361	156	180	0.966	0.160
	2	166.598	6.316	152	178	0.979	0.052
BMI	1	25.086	4.905	17.6	44.4	0.899	<0.001
	2	25.834	5.800	17.4	44.5	0.908	<0.001
Miscarriages	1	0.327	0.625	0	3	0.569	<0.001
	2	0.189	0.535	0	3	0.399	<0.001
Births	1	1.122	1.111	0	3	0.781	<0.001
	2	1.172	1.204	0	9	0.747	<0.001
Lesion size in mm (subjective size during hysteroscopy)	1 (*n* = 111)	8.400	7.092	2	20	0.862	0.236
	2 (*n* = 5)	12.072	5.900	3	30	0.917	<0.001
Lesion size in mm (objective size during ultrasound)	1 (*n* = 97)	14.600	11.118	4	35	0.845	0.050
	2 (*n* = 10)	12.031	5.018	4	25	0.932	<0.001
Procedure time	1	17.143	5.792	10.0	30.0	0.895	<0.001
	2	18.459	6.846	10.0	45.0	0.876	<0.001

**Table 6:** VAS perioperative pain.

	VAS: Pain		
	All	Hysteroscopy	
		Diagnostic	Operative
Procedure time	0.102	0.155	0.076
Lesion size in mm (subjective size during hysteroscopy)	0.213 *	−0.889 *	0.240 *
Lesion size in mm (objective size during ultrasound)	0.190	0.013	0.237 *

* *p* < 0.05.

**Table 7:** VAS vs. diagnosis.

	Diagnosis				KW (df = 3)	*p*
	Polyp (*n* = 93) *M* (SD)	Myomas (*n* = 19) *M* (SD)	Endometrial Hyperplasia/Abnormal Uterine Bleeding (*n* = 43) *M* (SD)	Infertility/Miscarriages (*n* = 16) *M* (SD)		
VAS: pain	2.88 (1.98)	3.00 (2.19)	2.79 (1.93)	2.26 (1.55)	0.289	0.962

**Table 8:** VAS vs. hysteroscopy.

	Hysteroscopy		UM-W	*p*
	Operative (*n* = 122) *M* (SD)	Diagnostic (*n* = 49) *M* (SD)		
VAS: pain	2.90 (2.01)	2.69 (1.78)	0.944	0.938

**Table 9:** VAS vs. depression.

	Depression: Declaration		UMW	*p*
	No (*n* = 148) *M* (SD)	Yes (*n* = 23) *M* (SD)		
VAS: pain	2.74 (1.86)	3.52 (2.37)	1417	0.19

**Table 10:** Two-factor ANCOVA for depression declaration and hysteroscopy type.

			95% Confidence Interval	
Hysteroscopy	Depression: Declaration	*M* (SD)	Lower Limit	Upper Limit
Diagnostic	No	2.80 (0.288)	2.226	3.36
	Yes	1.80 (0.856)	0.111	3.49
Operative	No	2.71 (0.188)	2.341	3.08
	Yes	4.00 (0.451)	3.110	4.89

**Table 11:** Beck Depression Inventory score.

	Depression				KW (df = 3)	*p*
	None (*n* =138) *M* (SD)	Mild (*n* = 20) *M* (SD)	Moderate (*n* = 6) *M* (SD)	Severe (*n* = 7) *M* (SD)		
VAS: pain	2.82 (2.00)	2.85 (1.64)	2.83 (1.17)	3.29 (2.29)	0.0914	0.949
	None (*n* = 138) *M* (SD)		Present (*n* = 33) *M* (SD)		KW (df = 1)	
	2.82 (2.00)		2.94 (1.69)		0.510	0.475

**Table 12:** Descriptive statistics.

	*M*	SD	Min.	Max.	*W*	*p*
VAS: pain	2.84	1.94	0	9	0.914	<0.001
Beck Depression Inventory	7.06	7.47	0.00	39.0	0.804	<0.001
Toronto Alexithymia Scale	46.25	12.12	21.00	76.0	0.985	0.064
Emotional Processing Scale	74.87	44.32	0.00	225.0	0.971	0.002

**Table 13:** Correlation coefficients between pain and emotional variables in three groups of patients.

	VAS: Pain		
	All	Operative	Diagnostic
Depression	0.011	0.046	−0.093
Alexithymia	0.197 **	0.168	0.274 *
Difficulty Identifying Feelings	0.262 ***	0.221 *	0.360 *
Difficulty Describing Feelings	0.114	0.097	0.155
Externally Oriented Thinking	0.044	0.039	0.056
Emotional processing	0.111	0.141	0.011
Suppression	0.102	0.147	−0.011
Signs of unprocessed emotion	0.154 **	0.209 *	−0.003
Unregulated emotion	0.032	0.048	−0.024
Avoidance	0.088	0.095	0.066
Impoverished emotional experience	0.105	0.115	0.059

* *p* < 0.05, ** *p* < 0.01, *** *p* < 0.001.

## Data Availability

The data presented in this study are available on request from the corresponding author.
